# Psychometric properties of an Arabic translation of the multidimensional assessment of interoceptive awareness (MAIA-2) questionnaire in a non-clinical sample of Arabic-speaking adults

**DOI:** 10.1186/s12888-023-05067-2

**Published:** 2023-08-09

**Authors:** Feten Fekih-Romdhane, Diana Malaeb, Mirna Fawaz, Nancy Chammas, Michel Soufia, Sahar Obeid, Souheil Hallit

**Affiliations:** 1 The Tunisian Center of Early Intervention in Psychosis, Department of psychiatry “Ibn Omrane”, Razi hospital, 2010 Manouba, Tunisia; 2 Faculty of Medicine of Tunis, Tunis El Manar University, Tunis, Tunisia; 3 College of Pharmacy, Gulf Medical University, Ajman, United Arab Emirates; 4 School of Pharmacy, Lebanese International University, Beirut, Lebanon; 5 Faculty of Health Sciences, Beirut Arab University, Tareek Al Jadida, Afeef Al Tiba, 1105 Beirut, Lebanon; 6 School of Medicine and Medical Sciences, Holy Spirit University of Kaslik, P.O. Box 446, Jounieh, Lebanon; 7 Social and Education Sciences Department, School of Arts and Sciences, Lebanese American University, Jbeil, Lebanon; 8 Psychology Department, College of Humanities, Effat University, 21478 Jeddah, Saudi Arabia; 9 Applied Science Research Center, Applied Science Private University, Amman, Jordan; 10 Research Department, Psychiatric Hospital of the Cross, Jal Eddib, Lebanon

**Keywords:** Interoceptive awareness, Interoception, MAIA-2, Psychometric properties, Arabic

## Abstract

**Background:**

Interoception refers to processes through which the nervous system identifies, analyzes, and integrates the information generated by the physiological state of the body (e.g., from internal organs such as the stomach, heart, or lungs). Despite its potential interest for clinical research and its wide use globally, no Arabic adaptation and validation of the Multidimensional Assessment of Interoceptive Awareness (MAIA-2) questionnaire exists to date. The goal of this study was to examine the psychometric properties of an Arabic translation of the MAIA-2 in a sample of Arabic-speaking community adults from Lebanon. We hypothesized that the Arabic version of the MAIA-2 would yield adequate internal consistency coefficients; the 8-factor structure model would show a good fit to our data, with measurement invariance and good convergent validity.

**Method:**

The Arabic adaptation of the MAIA-2 was developed using the forward–backward translation method. A non-clinical sample of Arabic-speaking adults (n = 359, 59.9% females, mean age = 22.75 years (SD = 7.04)) took part of this validation study. To check if the model was adequate, several fit indices were calculated: the normed model chi-square (χ²/df), the Steiger-Lind root mean square error of approximation (RMSEA), the Tucker-Lewis Index (TLI) and the comparative fit index (CFI). Values ≤ 3 for χ²/df, and ≤ 0.08 for RMSEA, and 0.90 for CFI and TLI indicate good fit of the model to the data.

**Results:**

Confirmatory Factor Analyses corroborated the validity of the original 8-factor structure of the MAIA-2 [χ^2^/df = 1603.86/601 = 2.67, RMSEA = 0.068 (90% CI 0.064, 0.072), SRMR = 0.058, CFI = 0.903, TLI = 0.892]. Reliability estimates in our sample revealed good internal consistency, with McDonald’s ω coefficients for the subscales ranging from 0.86 to 0.93. Our analyses also revealed measurement invariance of the Arabic MAIA-2 for gender. No statistically significant difference between men and women in all dimensions, except for the not worrying and attention regulation subscales where men scored significantly higher than women. Finally, the Arabic MAIA-2 dimensions showed positive correlations with the intuitive eating dimension “Reliance on Hunger and Satiety Cues”, thus providing support for convergent validity.

**Conclusion:**

We contribute the literature by providing the first Arabic adaptation and validation of a measure assessing the multidimensional construct of self-reported interoception. The Arabic MAIA-2 demonstrated good psychometric properties. We thus preliminarily recommend its use to measure the interoceptive awareness construct among Arabic-speaking communities worldwide.

## Introduction

Interoception refers to processes through which the nervous system identifies, analyzes, and integrates the information generated by the physiological state of the body (e.g., from internal organs such as the stomach, heart, or lungs) [1], thus allowing for “a moment-by-moment mapping of the body’s internal landscape across conscious and unconscious levels” [2] (p. 501). Interoceptive awareness refers to the multidimensional aspect of the conscious level of interoception that can be assessed through self-report measures [3, 4]. Interoceptive signals have been involved in several physiological and psychological processes, such as maintaining homeostasis [5], shaping cognitions, emotion regulation, self-awareness, time perception [6–10], as well as guiding our eating patterns and behaviours [11]. Interoceptive awareness influences our ability to eat intuitively, as it allows people to sense feelings of fullness and hunger [12, 13]. Intuitive eating was also proposed to “enhance interoceptive awareness or remove the obstacles to perceiving and responding to felt sensations in the body” ([12], p. 4). It is thus proposed that, if an individual has impaired sensitivity to interoceptive signals, he/she may be less adept to acknowledge physical hunger and satiety cues [11]. In sum, interoceptive awareness appears to be closely connected to intuitive eating.

Overall, interoceptive abnormalities have been incriminated in the etiology and treatment of a range of mental problems [2], including hypochondriasis, somatization, hypervigilance, anxiety [14], and disordered eating [15]. Accordingly, novel and promising treatment approaches targeting the dysfunctioning interoceptive system have been developed [2, 16–18]. For example, a narrative review focusing on intuitive eating interventions [15] indicated that dieting approaches disrupt interoceptive awareness, which hampers, in turn, individuals’ ability to become intuitive eaters and trust their internal signals. The review concluded that enhancing one’s interoceptive awareness may help them better understand and make meaning of internal cues (i.e. become a more efficient intuitive eater) [15]. More recently, different styles of interoceptive bodily awareness, i.e. mindful-based, have attracted the attention of researchers in the field of behavioral and integrative neuroscience [19, 20].

For all these reasons, there has been a steady growth in the amount of literature related to interoception during the last decade. As such, both objective and subjective measurements have been developed in the attempt to evolve research in this area. One of the most widely used objective measures for the introception construct, i.e. heart beat detection and counting tasks, have been questioned in their validity [21, 22]; and have proven to not reflect the clinically relevant variations of interoceptive abilities [23, 24]. These measures have also been shown to have limited capability of capturing the rich facets of the interoceptive construct [25]. Given these limitations, a particular focus has been placed on self-report measures of interoception, such as older brief Private Body Consciousness Scale (PPCS) [26], the Body Awareness Questionnaire (BAQ) [27], and the Body Perception Questionnaire (BPQ) [28]. However, these measures either cover narrow aspects of interoception, or focus on anxiety-driven symptoms [29]. One measure that enables the self-report assessment of multiple dimensions of interoception while differentiating between beneficial and maladaptive attention styles toward the body is the Multidimensional Assessment of Interoceptive Awareness (MAIA) [30, 31].

The MAIA is a 32-item questionnaire divided into eight subscales: (1) Noticing (4 items, awareness of neutral, pleasant, and uncomfortable bodily sensations); (2) Not-Distracting (3 items, tendency to either ignore or acknowledge body sensations of discomfort or pain); (3) Not-Worrying (3 items, capacity to maintain emotional balance despite sensations of discomfort or pain); (4) Emotional Awareness (5 items, awareness regarding the relation between bodily and emotional states); (5) Attention Regulation (7 items, capability to control or sustain attention towards body sensations); (6) Body Listening (3 items, ability to actively listen to one’s body sensations for insight); (7) Self-Regulation (4 items, ability to alleviate distress by using attention toward body sensations); and (8) Trusting (3 items, experience one’s body sensations as trustworthy and safe information sources) [30].

Since its publication in 2012, the MAIA has been adapted to different cultural groups and translated into over 20 languages, including German [25, 32], Italian [33], Spanish [34], Korean [35], Chinese [36], Persian [37], Lithuanian [38], Portuguese [39], Japanese [40, 41], and Malay [42]. It has also been validated in both clinical [43–45] and nonclinical [25, 33–35, 37–40] populations. Most of these linguistic versions of the original MAIA have generally demonstrated acceptable psychometric properties. At the same time, important shortcomings have been identified. Both the original [30] and other cross-cultural validations [25, 33, 34, 36, 46] of the MAIA revealed lower internal consistency of two subscales, i.e. Not-Worrying and Not-Distracting. One of the reasons that led to the low internal consistency reliability of these two three-item subscales is the use of Cronbach alpha which is sensitive to the items number [47]. In addition, a number of previous translational studies have failed to reproduce the original eight-factor structure of the MAIA [35, 38–42]. To overcome these gaps, Mehling et al. developed an updated 37-item version of the scale, i.e. the MAIA-2, which showed improved psychometric qualities [3].

This modified version has been translated to languages other than English, which generally supported its psychometric qualities; whereas the adequacy of its factorial structure remains disputable and inconclusive. For instance, the German MAIA-2 revealed appropriate reliability of the eight dimensions of the scale (range of ω: 0.70-0.90) in a sample of hospitalized patients with major depressive disorder [48]. Similarly, the French translation of MAIA-2 demonstrated satisfactory internal consistency, reliability over time, and construct validity in a community sample of French speaking adults; however, only six-factors were retained in the final model after excluding Not-Distracting and Not-Worrying factors [49]. The Chinese MAIA-2 version could retain only 31 items and a seven-factor model, while excluding the original MAIA scale “Noticing”; but good reliability (Cronbach’s alpha ranged from 0.656 to 0.838 for total scale and all seven subscales) and convergent/discriminant validity were demonstrated [50]. The Turkish validation study of the MAIA-2 demonstrated good reliability and validity of the scale; however, authors were compelled to make some modifications (e.g., addition or deletion of items) and were, therefore, only able to retain six dimensions with proper factor loadings while removing the Notice and Self-regulation factors [51]. It is therefore obvious that the factorial structure of the MAIA-2 still needs to be empirically explored and confirmed in different samples and contexts.

Furthermore, there is consistent evidence that females tend to more often notice bodily sensations, better understand how a bodily sensation may relate to an emotional state, perceive their body as less safe, experience more pain- or discomfort-related emotional distress, and are less accurate while detecting their heartbeats than males [52]. It is worth noting, however, that the original validation [30] as well as most of the previous linguistic validations of the MAIA involved samples largely dominated by females, and did not perform measurement invariance nor gender comparisons (e.g., Polish validation − 100% females [46]; Italian − 91% [33]; Chilean − 77% [34]; German, 68% [25]). Regarding gender comparisons, Da Costa Silva et al. [49] found that females exhibited higher levels of MAIA-2 Noticing, Emotional Awareness, and Body Listening dimensions compared to males; while males displayed greater Trusting scores than females. As for cross-gender invariance, we could find only two studies supporting this psychometric property, one among US youth [53] and another one among Malaysian adults [42].

Despite its potential interest for clinical research and its wide use globally, none of the self-report measures of the interoception construct is available in the Arabic language to the best of our knowledge. In particular, no Arabic adaptation and validation of the MAIA-2 exist to date. As such, no epidemiological prevalence data regarding Interoceptive Awareness can be found from Arab countries and the broader Arabic-speaking communities. Objective measures, such as heartbeat detection task, may be cost-burdening and not affordable for researchers working in Arab countries. Self-report measures offer multiple advantages, including easiness of administration and low cost. This could be potentially beneficial in Arab settings where there are deficits in the availability of mental health workforce [54–56].

In this context, the goal of this study was to examine the psychometric properties of an Arabic translation of the MAIA-2 in a sample of Arabic-speaking community adults from Lebanon in terms of factorial structure, composite reliability, and measurement invariance across gender groups. We hypothesized that: (1) the Arabic version of the MAIA-2 would yield adequate internal consistency coefficients; (2) the updated Mehling et al.’ s 8-factor structure model would show a good fit to our data; (3) cross-gender measurement invariance would be demonstrated at the metric, configural, and scalar levels; and (4) convergent validity. Convergent validity was tested through showing that MAIA-2 scores correlate to a relevant construct, i.e. intuitive eating, in the theoretically expected way. In particular, we hypothesized that if both interoceptive awareness (i.e. awareness of stimuli coming from one’s own body [6]) and intuitive eating (i.e. recognizing and responding to bodily sensations of hunger/fullness [12]) reflect the construct of body awareness, then participants felt engaged by internal body signals are expected to display positive correlations between these two constructs.

## Methods

### Participants

Three hundred fifty-nine participants participated in this study, with a mean age of 22.75 ± 7.04 years (age range 18–58) and 59.9% females. Other descriptive statistics of the sample can be found in Table 1.

**Table 1:** Sociodemographic and other characteristics of the participants (n = 359)

Variable	n (%)
Sex	
Male	144 (40.1%)
Female	215 (59.9%)
Marital status	
Single	331 (92.2%)
Married	28 (7.8%)
	**Mean ± SD**
Age (years)	22.75 ± 7.04
Body Mass Index (kg/m^2^)	24.12 ± 5.13
Household crowding index (person/room)	1.28 ± 1.92
Noticing	8.82 ± 4.56
Not distracting	16.41 ± 6.50
Not worrying	12.91 ± 2.73
Attention regulation	16.13 ± 7.74
Emotional awareness	12.51 ± 6.04
Self-regulation	9.10 ± 4.68
Body listening	6.45 ± 3.51
Trusting	7.47 ± 3.81
Reliance on Hunger and Satiety Cues	9.32 ± 3.22

### Translation procedure

The forward and backward translation method was applied to the interoceptive awareness scale following international guidelines [57]. The English version was translated to Arabic by a Lebanese translator who was completely unrelated to the study. Afterwards, a Lebanese psychologist with a full working proficiency in English, translated the Arabic version back to English. The initial and translated English versions were compared to detect and later eliminate any inconsistencies by a committee composed of the research team and the two translators [58, 59]. A pilot study was conducted on 20 persons before the start of the official data collection to make sure all questions are well understood; no changes were done consequently.

### Measures

#### The MAIA-2

This measures assesses the multifaceted aspects of self-reported interoceptive awareness through 37 items and eight dimensions (i.e., Noticing, Not-Distracting, Not-Worrying, Attention Regulation, Emotional Awareness, Self-Regulation, Body Listening, and Trusting) [3]. Items are scored on a six-point Likert scale ranging from 0 (never) to 5 (always), with higher scores referring to greater beneficial self-reported interoceptive awareness.

#### The intuitive eating scale-2 (IES-2)

This is a 23-item scale composed of four dimensions (i.e., Unconditional Permission to Eat, Eating for Physical Rather than Emotional Reasons, Reliance on Hunger and Satiety Cues, and Body-Food Choice Congruence) [60]. For the purposes of the present study, only the “Reliance on Hunger and Satiety Cues” dimension was used. In the Arabic validated version of the IES-2 [61], this subscale contains three items, and reflects trusting one’s own internal satiety and hunger cues and relying on them to guide eating behaviours. Each item is rated on a five-point scale ranging from 1 (strongly disagree) to 5 (strongly agree). Higher scores reflect greater reliance on internal body cues (McDonald’s ω = 0.88).

***Demographics***. Participants were asked to provide their demographic details consisting of age, sex, self-report weight and height to calculate the Body Mass Index (BMI), marital status and the Household Crowding Index (HCI); the latter reflecting the socioeconomic status of the family [62], is the ratio of the number of persons living in the house over the number of rooms in it (excluding the kitchen and the bathrooms).

### Procedures

All data were collected via a Google Form link, between September and November 2022. The project was advertised on social media and included an estimated duration. The research team approached people and asked them to fill the survey; those who accepted were asked to forward the link to other people they might know, explaining the snowball sampling technique followed. Inclusion criteria for participation included being of a resident and citizen of Lebanon of adult age (aged ≥ 18 years). Excluded were those who refused to fill out the questionnaire. Internet protocol (IP) addresses were examined to ensure that no participant took the survey more than once. After providing digital informed consent, participants were asked to complete the instruments described above, which were presented in a pre-randomised order to control for order effects. The survey was anonymous and participants completed the survey voluntarily and without remuneration [63].

### Analytic strategy

#### Confirmatory factor analysis

There were no missing responses in the dataset. We used data from the total sample to conduct a CFA using the SPSS AMOS v.29 software. The minimum sample size to conduct a confirmatory factor analysis ranges from 3 to 20 times the number of the scale’s variables [64]. Therefore, we assumed a minimum sample of 240 participants needed to have enough statistical power based on a ratio of 20 participants per one item of the scale, which was exceeded in our sample. Our intention was to test the original model of the MAIA-2 scores (i.e., eight-factor model). Normality was verified since the skewness and kurtosis values for each item of the scale varied between − 1 and + 1 [65]. Parameter estimates were obtained using the maximum likelihood method and fit indices. To check if the model was adequate, several fit indices were calculated: the normed model chi-square (χ²/df), the Steiger-Lind root mean square error of approximation (RMSEA), the Tucker-Lewis Index (TLI) and the comparative fit index (CFI). Values ≤ 3 for χ²/df, and ≤ 0.08 for RMSEA, and 0.90 for CFI and TLI indicate good fit of the model to the data [66]. The absence of multicollinearity was verified through tolerance values > 0.2 and variance inflation factor (VIF) values < 5. Multivariate normality was not verified at first (Bollen-Stine bootstrap p = .004); therefore we performed non-parametric bootstrapping procedure (available in AMOS).

#### Gender invariance

To examine gender invariance of MAIA scores, we conducted multi-group CFA [67] using the total sample. Measurement invariance was assessed at the configural, metric, and scalar levels [68]. Configural invariance implies that the latent scales variable(s) and the pattern of loadings of the latent variable(s) on indicators are similar across gender (i.e., the unconstrained latent model should fit the data well in both groups). Metric invariance implies that the magnitude of the loadings is similar across gender; this is tested by comparing two nested models consisting of a baseline model and an invariance model. Lastly, scalar invariance implies that both the item loadings and item intercepts are similar across gender and is examined using the same nested-model comparison strategy as with metric invariance [67]. Following the recommendations of Cheung and Rensvold (2002) [69] and Chen (2007) [67], we accepted ΔCFI ≤ 0.010 and ΔRMSEA ≤ 0.015 or ΔSRMR ≤ 0.010 (0.030 for factorial invariance) as evidence of invariance [63].

#### Further analyses

Composite reliability was assessed using McDonald’s ω, with values greater than 0.70 reflecting adequate composite reliability [70]. McDonald’s ω was selected as a measure of composite reliability because of known problems with the use of Cronbach’s α (e.g., [71]). All MAIA-s subscales scores were considered normally distributed according to their skewness and kurtosis values varying between ± 1 [65]. Consequently, the Pearson test was used to correlate those scores with age, BMI and reliance on hunger and satiety cues score.

## Results

### Confirmatory factor analysis of the MAIA-2 scale

CFA indicated that fit of the eight-factor model of the MAIA scale (model 1) [3] was acceptable: χ^2^/df = 1603.86/601 = 2.67, RMSEA = 0.068 (90% CI 0.064, 0.072), SRMR = 0.058, CFI = 0.903, TLI = 0.892.

The fit indices of the seven-factor model of the Chinese version of the scale (model 2) [50] were as follows: χ^2^/df = 1278.61/413 = 3.10, RMSEA = 0.077 (90% CI 0.072, 0.081), SRMR = 0.058, CFI = 0.902, TLI = 0.890.

The fit indices of the six-factor model of the French version of the scale (model 3) [49] were as follows: χ^2^/df = 855.11/284 = 3.01, RMSEA = 0.075 (90% CI 0.069, 0.081), SRMR = 0.046, CFI = 0.925, TLI = 0.914. The standardised estimates of factor loadings were all adequate for all models (see Table 2).

**Table 2:** Factor Loadings Derived from the Confirmatory Factor Analysis of the 8-, 7- and 6-factor models of the Multidimensional Assessment of Interoceptive Awareness-2 in the total sample

	8-factor	7-factor	6-factor
Factor 1: Notice			
1	0.76		0.77
2	0.84		0.84
3	0.78		0.79
4	0.75		0.73
Factor 2: Not distracting			
5	0.59	0.59	
6	0.77	0.77	
7	0.76	0.76	
8	0.78	0.78	
9	0.86	0.87	
10	0.82	0.82	
Factor 3: Not worrying			
11	0.78	0.76	
12	0.71	0.71	
13	0.74	0.72	
14	0.54	0.57	
15	0.67		
Factor 4: Attention regulation			
16	0.70		0.70
17	0.83	0.82	0.83
18	0.83	0.83	0.83
19	0.84	0.84	0.84
20	0.79	0.79	0.79
21	0.82	0.82	0.82
22	0.84	0.84	0.84
Factor 5: Emotional awareness			
23	0.76	0.75	0.76
24	0.76	0.76	0.76
25	0.83	0.84	0.83
26	0.83	0.83	0.83
27	0.84	0.84	0.84
Factor 6: Self-regulation			
28	0.81	0.81	0.81
29	0.83	0.83	0.83
30	0.83	0.83	0.83
31	0.83	0.83	0.83
Factor 7: Body listening			
32	0.81	0.81	0.81
33	0.88	0.88	0.88
34	0.84	0.84	0.84
Factor 8: Trust			
35	0.83	0.83	0.82
36	0.90	0.90	0.90
37	0.89	0.89	0.89

### Gender invariance of the MAIA-2 scale

As reported in Table 3, we were able to show the invariance across gender at the configural, metric, and scalar levels. No statistically significant difference between men and women in all dimensions, except for the not worrying and attention regulation subscales where men scored significantly higher than women (Table 4).

**Table 3:** Measurement Invariance of the Multidimensional Assessment of Interoceptive Awareness-2 scale Across Gender in the total sample

Model	χ²	*df*	CFI	RMSEA	SRMR	Model Comparison	Δχ²	ΔCFI	ΔRMSEA	ΔSRMR	Δ*df*	*p*
Configural	2672.65	1202	0.864	0.059	0.076							
Metric	2694.75	1231	0.864	0.058	0.077	Configural vs. metric	22.1	0 < 0.001	0.001	0.001	29	0.816
Scalar	2742.78	1260	0.863	0.057	0.077	Metric vs. scalar	48.03	0.001	0.001	< 0.001	29	0.014

*Note.* CFI = Comparative fit index; RMSEA = Steiger-Lind root mean square error of approximation; SRMR = Standardised root mean square residual

**Table 4:** Comparison between sexes in terms of the Multidimensional Assessment of Interoceptive Awareness-2 scale and subscales scores in the total sample

	Noticing	Not distracting	Not worrying	Attention regulation	Emotional awareness	Self-regulation	Body listening	Trust
Males	8.37 ± 4.46	15.77 ± 6.46	13.29 ± 2.88	17.47 ± 7.16	12.47 ± 5.74	9.22 ± 4.42	6.65 ± 3.42	7.78 ± 3.62
Females	9.12 ± 4.61	16.84 ± 6.50	12.66 ± 2.60	15.24 ± 8.00	12.53 ± 6.24	9.02 ± 4.85	6.32 ± 3.56	7.26 ± 3.93
t	1.527	1.534	2.156	2.695	-0.096	0.381	0.879	1.277
*p*	0.128	0.126	**0.032**	**0.007**	0.923	0.704	0.380	0.202
95% CI of the difference	-1.71; 0.22	-2.44; 0.30	0.06; 1.21	0.60; 3.85	-1.34; 1.22	-0.80; 1.18	-0.41; 1.07	-0.28; 1.33
Effect size	0.165	0.165	0.229	0.293	0.010	0.043	0.094	0.137

Numbers in bold indicate significant *p* values. Ranges of scores: Notice [0–20], not distracting [0–30], not worrying [0–25], attention regulation [0–35], emotional awareness [0–25], self-regulation [0–20], body listening [0–15] and trust [0–15]

### Composite reliability of the MAIA-2 scale

Composite reliability of scores was adequate in the total sample for the notice (ω = 0.86), not distracting (ω = 0.90), not worrying (ω = 0.82), attention regulation (ω = 0.93), emotional awareness (ω = 0.90), self-regulation (ω = 0.90), body listening (ω = 0.88) and trust (ω = 0.90).

### Convergent validity of the MAIA-2 scale

To assess the validity of the MAIA-2 scores, we examined bivariate correlations with reliance on hunger and satiety cues in the present study using the total sample. Higher reliance on hunger and satiety cues scores was significantly associated with more noticing, attention regulation, emotion awareness, self-regulation, and body listening and trusting (Table 5).

**Table 5:** Correlations of the Multidimensional Assessment of Interoceptive Awareness-2 subscales scores with the other measures in the total sample

	1	2	3	4	5	6	7	8	9	10	11
1. Noticing	1										
2. Not-distracting	-0.59***	1									
3. Not-worrying	-0.25***	0.22***	1								
4. Attention regulation	0.58***	-0.59***	-0.15**	1							
5. Emotional awareness	0.70***	-0.63***	-0.23***	0.77***	1						
6. Self-regulation	0.50***	-0.51***	-0.14**	0.74***	0.77***	1					
7. Body listening	0.51***	-0.47***	-0.18**	0.67***	0.72***	0.75***	1				
8. Trusting	0.49***	-0.47***	-0.10	0.65***	0.61***	0.65***	0.74***	1			
9. Reliance on Hunger and Satiety Cues	0.26***	-0.19***	0.02	0.31***	0.23***	0.28***	0.35***	0.37***	1		
10. Age	-0.01	-0.04	0.002	0.01	0.01	-0.03	-0.01	0.01	-0.07	1	
11. Body Mass Index	-0.11*	0.04	0.01	-0.11*	-0.15**	-0.13*	-0.16**	-0.18**	0.003	0.14**	1

*p < .05; **p < .01; ***p < .001

## Discussion

We contribute the literature by providing the first Arabic adaptation and validation of a measure assessing the multidimensional construct of self-reported interoception. As expected, we found that the Arabic MAIA-2 yielded excellent internal consistency reliability, and confirmed the 8-factor structure with invariance by gender, and showed good convergent validity in relation to the intuitive eating dimension “Reliance on Hunger and Satiety Cues”.

CFA further corroborated the factorial validity of the original 8-, 7- and 6-factor structures of the MAIA-2 in our non-clinical sample. To our knowledge, only a very few dimensional examinations of the MAIA-2 are available so far. In line with our findings, the German validation was able to retain the originally proposed eight factors [48], as well as the Chinese validation of the MAIA-2 that resulted in a 7-factor model [72] and the French [49] and Turkish [51] validations, which retained only six-factors. In summary, our results demonstrate the goodness of the Mehling et al.’s eight-factor structure for the MAIA-2 and support its use for the purpose of assessing eight distinct Interoceptive Awareness dimensions.

Reliability estimates in our sample revealed good internal consistency, with McDonald’s ω coefficients for the eight subscales ranging from 0.86 to 0.93. The original validation found Cronbach alphas ranging from 0.64 to 0.83 for the eight scales in a large sample of native English-speaking adults from the UK, thus highlighting that two subscales were below the standard criterion of 0.70 (Not Worrying and Noticing) [3]. The Chinese version of the MAIA-2 yielded low Cronbach’s alpha values for the Noticing subscale resulting in its removal; while all other subscales showed alphas varying from 0.656 to 0.838 [72]. We note that most of the previous validation studies relied on Cronbach’s α [42], which might possibly lead to risk for bias. Thus, a major strength of the present study is the use of McDonald’s ω [73], which has proven to estimate more reliably the internal consistency of psychological scales [32, 70]. Recently, a German study explored the reliability of the MAIA-2 in a clinical sample of depressed patients undergoing hospital treatment, and showed appropriate post-treatment reliabilities for the eight subscales (ω ≥ 0.70), whereas pre-treatment reliabilities were questionable for the Not-Distracting and Noticing scales (ω < 0.70) [48]. The large discrepancy in the literature data calls for additional cross-cultural validations to further confirm the reliability and factorial validity of the MAIA-2.

Our analyses also revealed measurement invariance of the Arabic MAIA-2 for gender. Confirming the invariance of the scale by gender groups allows future reliable comparisons of mean scores between males and females. In this regard, our results indicated that males scored significantly higher in worrying and attention regulation subscales than female participants. No statistical differences were observed in other MAIA dimensions between genders. These findings are inconsistent with previous literature generally showing that females tend to more often notice body sensations, and exhibit more worries related to sensations of discomfort or pain compared to males [52]. Given the sufficient available evidence that Interoceptive awareness vary across genders, it has been strongly recommended that gender be accounted for when performing subjective evaluation of the Interoceptive awareness construct disorders related to emotional/mood states and/or bodily sensations [52]. One important consideration, however, is that a very few studies have investigated cross-gender invariance prior to performing between-gender comparisons [52]. The vast majority of available linguistic validations of both original and modified versions of the scale did not perform gender invariance or gender comparisons of latent MAIA-2 means (e.g., German [48] and Chinese [50] MAIA-2). We could find only two studies that investigated measurement invariance across genders. The first study found that the youth-adapted version of the MAIA was invariant at the scalar level across both gender groups in children and adolescents aged 7–17 years [53]. The second study found that the 8-factor model of the Malay version of the MAIA also showed full strict invariance across genders [42].

Finally, results showed that MAIA-2 scores correlated positively with the IES-2 “Reliance on Hunger and Satiety Cues” subscores, thus providing support for convergent validity of the Arabic MAIA-2. These findings are in line with previous literature suggesting that interceptive awareness is closely linked to intuitive eating [11, 15, 74], and that MAIA-2 and IES-2 assess related but distinct body awareness constructs. Results also provide more support to the clinical relevance of the Interoceptive Awareness construct in the Arab context. Indeed, the correlations of different facets of interoception with high levels of intuitive eating confirm the previous assumptions that interoception appears to be implicated in several psychological processes including disordered eating, and could be involved in their treatment [2, 15], including in Arab contexts.

### Study limitations

Some limitations need to be discussed. We referred to a cross-sectional design, which precluded the investigation of temporal consistency of the Arabic MAIA-2. Thus, future studies still need to examine the test-retest reliability of the scale. Other information regarding family and personal history of diseases and relevant mental health issues was not collected, which might have affected our findings. A selection bias is present since the response rate cannot be known. In addition, the web-based design attracted mostly young participants of female gender, which may limit the generalizability of our findings. It is of note, however, that like all Arab countries, Lebanon has one of the most youthful age structures in the world (around 60% of the population are under 25 years old) [75]. In 2021, about 28% of Lebanon’s total population were aged 0 to 14 years [76]. Other psychometric properties not conducted in this study (test-retest, convergent, divergent and discriminant validity, criterion related validity) are yet to be performed to confirm the stability of the Arabic MAIA-2 scores. In addition, factorial structure was only verified by the CFA; whereas the interoceptive awareness construct might have been impacted by the Arab cultural background [77]. Therefore, future studies need to further confirm the 8-factor structure model of the Arabic 37-item MAIA-2 by Exploratory Factor Analysis. Finally, cross-national validations in samples from different Arab countries are still needed to further confirm the validity of the scale in the broad Arabic-speaking community.

### Clinical implications

Our present findings bare some important clinical implications. Findings demonstrated that the Arabic version of the MAIA-2 is reliable, valid, and invariant across gender groups. This means that respondents of both genders interpreted items similarly; and that the latent means may be compared meaningfully between males and females in future research. We believe that making the Arabic MAIA-2 available may attract the attention of Arab researchers to the role of Interoceptive Awareness in mental health, and pave the way for future observational and experimental research in this area. Indeed, our study sheds light on the lack of research on this topic in Arab people; which is partly due to a lack of easy-to-use, economic, and valid instruments to assess this construct. We therefore call for national and cross-national research on Interoceptive Awareness involving Arab countries. Future international comparisons may help identify cultural characteristics of Interoception, and how it may interfere with health indicators in Arabic-speaking populations.

## Conclusion

Taken together, our findings suggest that the psychometric characteristics of the Arabic MAIA-2 are robust, at least in Lebanese community adults. Therefore, we preliminarily recommend its use to measure the interoceptive awareness construct among Arabic-speaking communities in Lebanon and other parts of the world. We believe that offering a reliable and valid Arabic version of the MAIA-2 will hopefully encourage research output on Interoception in Arab countries and various disciplinary backgrounds; and will have important implications for improving the quality of cross-cultural studies in this area.

## Data Availability

The datasets generated and/or analysed during the current study are not publicly available due to restrictions from the ethics committee but are available from the corresponding author on reasonable request.
